# A New Sensing Platform Based in CNF-TiO_2_NPs-Wax on Polyimide Substrate for Celiac Disease Diagnostic

**DOI:** 10.3390/bios15070431

**Published:** 2025-07-04

**Authors:** Evelyn Marín-Barroso, Maria A. Ferroni-Martini, Eduardo A. Takara, Matias Regiart, Martín A. Fernández-Baldo, Germán A. Messina, Franco A. Bertolino, Sirley V. Pereira

**Affiliations:** 1 Instituto de Química de San Luis (INQUISAL), Departamento de Química, Facultad de Química Bioquímica y Farmacia, Universidad Nacional de San Luis, Avenida Ejército de los Andes 950, San Luis D5700HHW, Argentina; evymarinbarroso@gmail.com (E.M.-B.); maferroni@unsl.edu.ar (M.A.F.-M.); mregiart@unsl.edu.ar (M.R.); mbaldo@unsl.edu.ar (M.A.F.-B.); messina@unsl.edu.ar (G.A.M.); 2 Consejo Nacional de Investigaciones Científicas y Técnicas (CONICET), Av. Rivadavia 1917, Buenos Aires C1033AAJ, Argentina; eatakara@unsl.edu.ar; 3 Instituto de Física Aplicada (INFAP), Departamento de Química, Facultad de Química Bioquímica y Farmacia, Universidad Nacional de San Luis, Avenida Ejército de los Andes 950, San Luis D5700HHW, Argentina

**Keywords:** immunosensor, nanomaterials, electrochemical, celiac disease diagnosis

## Abstract

Celiac disease (CD), a human leukocyte antigen-associated disorder, is caused by gluten sensitivity and is characterized by mucosal alterations in the small intestine. Currently, its diagnosis involves the determination of serological markers. The traditional method for clinically determining these markers is the enzyme-linked immunosorbent assay. However, immunosensors offer sensitivity and facilitate the development of miniaturized and portable analytical systems. This work focuses on developing an amperometric immunosensor for the quantification of IgA antibodies against tissue transglutaminase (IgA anti-TGA) in human serum samples, providing information on a critical biomarker for CD diagnosis. The electrochemical device was designed on a polyimide substrate using a novel solid ink of wax and carbon nanofibers (CNFs). The working electrode microzone was defined by incorporating aminofunctionalized TiO_2_ nanoparticles (TiO_2_NPs). The interactions and morphology of CNFs/wax and TiO_2_NPs/CNFs/wax electrodes were assessed through different characterization techniques. Furthermore, the device was electrochemically characterized, demonstrating that the incorporation of CNFs into the wax matrix significantly enhanced its conductivity and increased the active surface area of the electrode, while TiO_2_NPs contributed to the immunoreaction area. The developed device exhibited remarkable sensitivity, selectivity, and reproducibility. These results indicate that the fabricated device is a robust and reliable tool for the precise serological diagnosis of CD.

## 1. Introduction

Celiac disease (CD) is an autoimmune disorder generated in genetically predisposed individuals when they are exposed to gluten consumption. It mainly affects the gastrointestinal system through an abnormal immune response [1,2]. Human leukocyte antigens HLA-DQ2 and HLA-DQ8 are related to the expression of this disease [3]. Villous atrophy and crypt hyperplasia result from the lack of early diagnosis and treatment. These mucosal alterations cause malabsorption and, in the long term, predisposition to severe complications such as osteoporosis, infertility, and lymphoma [4]. For these reasons, a rapid and accurate diagnosis is of great relevance. Although serological tests have significantly improved, the gold standard test remains the duodenal biopsy [5]. This examination represents an invasive and troublesome procedure, especially for pediatric patients. In this sense, the use of serological markers is convenient. CD generates autoantibodies against transglutaminase (anti-TGA) and endomysium (anti-EMA) of the intestinal villi, as well as against external agents, such as gluten proteins (gliadin), among others [6]. The presence of anti-TGA and anti-EMA antibodies and their absence when maintaining a gluten-free diet are considered critical diagnostic tools [7]. The traditional method for clinically determining these serological markers is the enzyme-linked immunosorbent assay (ELISA) with optical detection [8]. However, the increasing demand for portable, simple, and sensitive diagnostic tools generates the need for sensing devices integrated with different detection systems.

Immunosensors meet these requirements and allow the obtaining of miniaturized and portable analysis systems [9]. Electrochemical transduction is also attractive due to its excellent attributes, simplicity, and low cost. Nevertheless, developing portable sensing devices with electrochemical detection forces us to look for new support materials [10]. Polyimide represents an interesting option as a support material due to its flexibility and excellent mechanical, thermal, and chemical resistance. Furthermore, it is accessible and allows the deposition of a great diversity of conductive materials for the electrode construction [11]. The most used conductive materials are those derived from carbon, such as graphite, graphene, carbon nanofibers, and carbon nanotubes [12]. Carbon nanofibers (CNFs) have excellent conduction properties due to their increased electroactive surface area, higher electron transfer, and molecule adsorption. Additionally, these materials have an irregular microarchitecture that is favorable for biomolecules immobilization. Titanium dioxide nanoparticles (TiO_2_NPs) also represent an attractive material due to their good biocompatibility, photo, and chemical stability [13,14]. This oxide exhibits high adsorption capacity, low toxicity, and production cost. Therefore, it is an adequate material for electrode modification with excellent compatibility with CNFs [15].

Wax printing has emerged as an adequate alternative to create patterns for electrochemical device fabrication [16]. This technique enables the deposition of solid wax inks on different substrates using wax standard printers. Due to its melting point and viscosity, wax generates compact solids by simply heating which represents an attractive strategy for designing conductive patterns on several surfaces for electrochemical device fabrication [17,18].

In this work, an immunosensor with amperometric detection was developed to quantify IgA anti-TGA present in human serum samples. The electrochemical device was designed on a polyimide substrate by depositing a new solid ink obtained from wax and CNFs. This new composite material allows for the printing of working, reference, and auxiliary electrode tracks on the polymeric surface. Finally, the working electrode microzone, where the immunoreaction takes place was defined by incorporating the nanocomposite TiO_2_NPs/CNFs/wax. The designed device showed an amperometric signal that directly corresponds to the anti-TGA concentration, demonstrating excellent sensitivity and robustness. To conclude, the newly developed immunosensor represents an effective analytical tool for the serological diagnosis of CD.

## 2. Materials and Methods

### 2.1. Reagents and Solutions

TGA from guinea pig liver, anti-human IgA conjugated antibody, bovine serum albumin (BSA), carbon nanofibers (CNFs graphitized, composed of conical platelets), catechol (Q), 3-Aminopropyltriethoxysilane (APTES), glutaraldehyde (25% aqueous solution), methanol, ethanol, hydrogen peroxide, titanium (IV) oxide, and chloroform were purchased from Sigma-Aldrich (St. Louis, MO, USA). Potassium chloride, sodium chloride, monobasic/dibasic sodium phosphate, potassium ferricyanide, and ferrocyanide were acquired from Merck (Darmstadt, Germany). All solutions were prepared with Milli-Q water. The polyimide film (Kapton^®^ polyimide film) was obtained from Dupont (Wilmington, DE, USA). The enzyme immunoassay for anti-TGA IgA antibodies by BioSystems (Buenos Aires, Argentina) was performed according to the manufacturer’s instructions. The kit contained six standards (0, 5, 10, 25, 50, 100 U mL^−1^) and positive and negative control. The reagents used in all experiments were of analytical reagent grade.

### 2.2. Instrumentation

Electrochemical measures were obtained with BAS 100 B/W (Bioanalytical Analyzer Electrochemical System, West Lafayette, IN, USA) and Autolab PGSTAT302N potentiostat/galvanostat (Metrohm Autolab B.V., Utrecht, The Netherlands). Electrodes with their delimited microzones were designed using CorelDraw 12 software and printed with a Xerox ColorQube 8870 printer from Xerox (Buenos Aires, Argentina), considering the dimensions of a commercial electrode. Cyclic voltammograms and amperometric measurements were obtained using a designed electrode with a working electrode diameter of 3.0 mm. Orion Research Inc. (Orion Research Inc., Cambridge, MA, USA) Model EA 940 equipped with a glass combination electrode was employed to measure the pH value of all solutions. The nanocomposite film morphology was studied by scanning electron microscopy LEO 1450VP (SEM) (Jena, Germany), and the crystalline structures and interactions were characterized using a Rigaku D-Max III C X-ray diffractometer (XRD) (Tokyo, Japan) and a Varian 640 infrared spectrometer (FTIR) (Palo Alto, CA, USA), respectively. Spectrometer Absorbance was measured using a BioTek Epoch microplate spectrophotometer model Take3 (Winooski, VT, USA). Purified water was employed in the preparation of all solutions and standards using a Millipore Milli-Q system (Bedford, MA, USA).

### 2.3. Surface Aminofunctionalization of TiO_2_NPs with APTES

For the bioaffinity support obtention, the surface of the TiO_2_NPs was modified with APTES solution in a ratio of 1:1. In the first stage, 1 mL of TiO_2_NPs and 0.4 g of APTES were added in 50 mL of ethanol solution and stirred for 24 h. Then, to remove the excess reagent, the obtained dispersion was washed twice with Milli-Q water and five times with methanol. Finally, the dispersion was dried at 80 °C for 24 h.

### 2.4. Synthesis of Nanostructured Conductor Wax Solid Ink

Two solid wax inks were prepared. The first (used for the printing of working tracks, reference, and auxiliary electrode) CNFs/wax solid ink was obtained by heating 1 g of wax to 90 °C, next adding 0.35 g of CNFs and stirring continuously for 1 h. The second (employed for design of the working electrode microzone) TiO_2_NPs/CNFs/wax solid ink was prepared by first resuspending 20 mg of amino-functionalized TiO_2_NPs in methanol. Next, 0.35 g of CNFs were added to the dispersion and the mixture was vigorously stirred for 1 h. The dispersion was then dried at 80 °C for 24 h. The resulting solid was mixed with 1 g of wax at 90 °C and stirred vigorously for 1 h.

### 2.5. Electrode System Design

**Figure 1:**
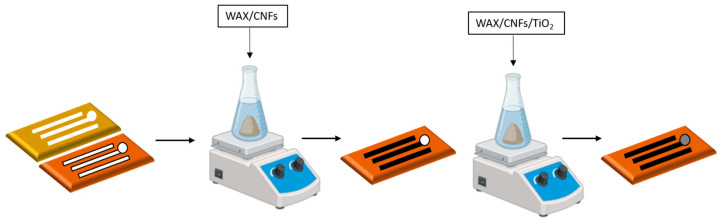
Design and fabrication of the three-electrode system using wax-printed hydrophobic barriers and selective application of CNFs/wax and TiO_2_NPs/CNFs/wax inks. Electrochemical activation was carried out in a buffer solution at pH 4.5 at −9 V for 10 s to prepare the working electrode.

The electrode system (three electrodes) was designed with Corel Draw 12. This design was transferred through the printing of hydrophobic barriers using a conventional wax printer (Xerox ColorQube 8870) and commercial wax, which delimited each electrode surface. Then, using a mask CNFs/wax, ink was applied to design the reference electrode, auxiliary electrode (track and electrode area), and working electrode track. In contrast, TiO_2_NPs/CNFs/wax was deposited in the working electrode microzone (Figure 1). The diameter of the working electrode was 3 mm. The Randles–Sevcik equation was used to calculate the electroactive surface area (Supplementary Material Section S1). Finally, the obtained electrode underwent an electrochemical activation process before use. The activation was performed by immersing the electrode in a buffer solution containing 0.5 mol L^−1^ acetic acid and sodium acetate at pH 4.5 at −9 V for 10 s.

### 2.6. TG Immobilization on TiO_2_NPs/CNFs/Wax and IgA Anti-TGA Measurement

The transglutaminase enzyme (TG) was covalently immobilized on the surface of aminofunctionalized TiO_2_NPs adding 5 µL of 0.20 mol L^−1^ glutaraldehyde solution in 0.1 mol L^−1^ sodium phosphate buffer pH 8.0 on the electrode surface at 25 °C for 2 h. After, a washing procedure was performed with 0.01 mol L^−1^ phosphate buffer pH 7.0. Later, 5 µL of a 10 μg mL^−1^ TGA enzyme solution in 0.01 mol L^−1^ phosphate buffer pH 7.0 was added to the working electrode area for 12 h at 4 °C (aldehyde groups of TiO_2_NPs reacted with amino moieties of TGA enzyme). Finally, a blocking step with 1% BSA for 5 min was carried out to avoid non-specific binding.

**Figure 2:**
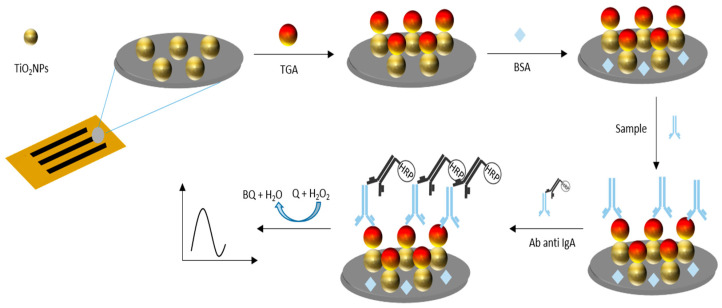
Schematic representation of the immobilization of TG onto TiO_2_NPs via glutaraldehyde crosslinking and subsequent IgA anti-TGA antibody detection. The electrochemical signal, generated by HRP-catalyzed oxidation of catechol, was proportional to the antibody concentration in serum samples.

IgA anti-TGA antibodies were determined using serum samples previously diluted 1:100 in 0.01 mol L^−1^ phosphate-buffer solution pH 7.0. Only 5 μL diluted samples were placed on the working electrode area, incubated at room temperature in the humidity chamber for 15 min, and washed with 0.01 mol L^−1^ phosphate buffer pH 7.0. Bound antibodies, which recognized immobilized TG, were quantified by adding 5 μL of anti- α human chain antibody conjugate with HRP for 5 min (dilution of 1:1000 in 0.01 mol L^−1^ sodium phosphate buffer, pH 7.0). Finally, to perform amperometric measurements, the electrochemical platform was exposed to the addition of 5 μL of a 1 mmol L^−1^ citrate-phosphate-buffer solution pH 5.0, applying a detection potential of −0.15 V. Once the background current stabilized, 5 μL of a substrate solution containing 1 mmol L^−1^ catechol (Q) and 1 mmol L^−1^ H_2_O_2_ were incorporated. The electrochemical signal response corresponding to the reduction current of o-benzoquinone (BQ) was measured at 60 s. The signal was proportional to the concentration of IgA anti-TGA antibodies in the samples (Figure 2).

## 3. Results

### 3.1. Characterization of the Designed Electrodes

**Figure 3:**
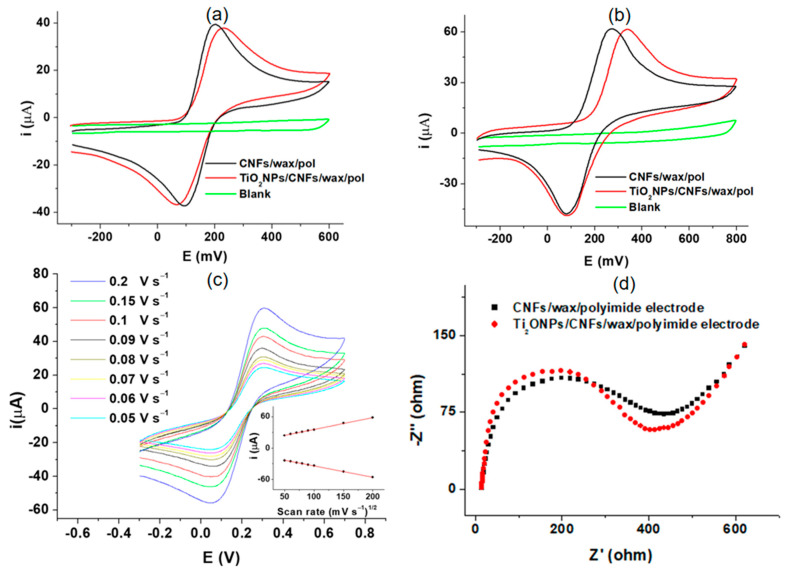
(**a**) shows the corresponding CVs of blank for CNFs/wax/polyimide electrode recorded in PBS solution 0.1 mol L^−1^ pH 6.50 (green line), CNFs/wax/polyimide electrode (black line), and TiO_2_NPs/CNFs/wax/polyimide electrode (red line) in a 1 mmol [Fe(CN)6]^4−/3−^ redox couple solution in PBS 0.1 mol L^−1^ pH 6.5; (**b**) exhibits CVs for the same electrode platforms in 1 mm L^−1^ of Q solution in a 1 mm L^−1^ citrate-phosphate buffer at pH 5.0; (**c**) shows the influence of scan rate on the peak current obtention; and (**d**) EIS measurements for the electrode platform before and after TiO_2_NPs incorporation.

The TiO_2_NPs/CNFs/wax/polyimide electrode was electrochemically characterized by cyclic voltammetry (CV) with the [Fe(CN)_6_]^4−/3−^ redox couple at 1 mmol in pH 6.50 PBS 0.1 mol L^−1^. The potential scan was performed from − 0.3 to 0.6 V at a speed of 0.075 V s^−1^ (Figure 3a). As expected, a single anodic and a single cathodic peak were obtained (39 μA at 0.20 V and −37 μA at 0.09 V, respectively) when the CNFs/wax/polyimide was used (black line). Including TiO_2_NPs on the electrode surface (TiO_2_NPs/CNFs/wax/polyimide electrode red line) did not result in significant changes in the peak currents (37 μA at 0.23 V and −37 μA at 0.06 V, respectively) due to its electrical inherent properties [19]. However, its incorporation improves the reaction area through the enhancement of immobilization capacity.

The proposed method for the determination of anti-TGA employs an electrochemical mediator (catechol(Q)), which is added to the revealing solution. Therefore, the behavior of this was also evaluated through CV on the TiO_2_NPs/CNFs/wax/polyimide electrode surface (Figure 3b). This study was conducted using a 1 mm L^−1^ Q solution in a 1 mm L^−1^ citrate-phosphate buffer at pH 5.0, as this pH value is optimal for enzymatic activity. A single anodic and cathodic peak corresponding to the transformation of Q to BQ and vice versa were obtained (61 μA at 0.27 V and −47 μA at 0.08 V, respectively) when the CNFs/wax/polyimide electrode was used (black line). For TiO_2_NPs/CNFs/wax/polyimide electrode (red line), no significant changes in peak current responses were observed, (61 μA at 0.33 V and −47 μA at 0.08 V, respectively) exhibiting a behavior consistent with Figure 3a [20]. It is important to take into account that the two electrochemical probes used in the evaluations ([Fe(CN)_6_]^4−^/^3−^ and Q; Figure 3a,b, respectively) exhibited different electrochemical behaviors and involved different numbers of electrons transferred (one and two electrons, respectively). Accordingly, a higher current is expected for the electrode exposed to the Q solution.

An evaluation of the influence of scan rate on peak current was performed to examine the electrochemical behavior of the TiO_2_NPs/CNFs/wax/polyimide electrode in [Fe(CN)_6_]^4−/3−^ redox couple at 1 mmol in pH 6.50 PBS 0.1 mol L^−1^ at a potential range from −0.4 to 0.8 V. The study enclosed a range of scan rates, varying from 0.05 to 0.2 V s^−1^ (Figure 3c). As can be appreciated, the oxidation and reduction peak currents show a linear correlation with the square of scan rate (Figure 3c inset) in the evaluated range (0.05 to 0.2 V s^−1^). The obtained results expose the existence of a fast electrochemical and diffusion-controlled process [21]. Later, electrochemical impedance spectroscopy (EIS) measurements were carried out using a [Fe(CN)_6_]^4−/3−^ solution (4 mM in KCl 0.1 mol L^−1^) to obtain additional information about the effect of TiO_2_NPs incorporation on the charge transfer process (Figure 3d). The Nyquist diagram shows that the incorporation of TiO_2_NPs to CNFs/wax/poliymide electrode (red diagram) leads to a slight change in the RCT compared to CNFs/wax/poliymide electrode without TiO_2_NPs (black diagram) from 408 Ω 389. In addition, the solution resistance (Rs) and the double-layer capacitance (Cdl) were calculated. The Rs were found to be 13.6 Ω. The Cdl values were 2.97 × 10^−5^ F in the absence of Ti (black diagram) and 1.42 × 10^−5^ F in the presence of Ti (red diagram). These results were obtained employing a Randles equivalent circuit, and are consistent with the behavior previously described for the CVs of Figure 3a.

**Figure 4:**
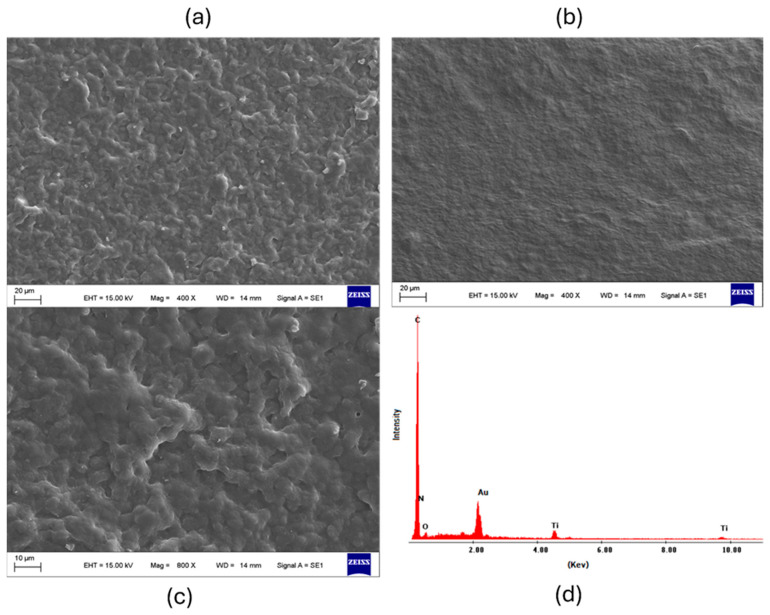
SEM images show the morphological changes on the wax/polyimide electrode surface after incorporating CNFs and TiO_2_NPs at different magnifications ((**a**) wax surface image, (**b**) TiO_2_NPs/CNFs/wax at 400×, (**c**) TiO_2_NPs/CNFs/wax at 800×) and (**d**) EDS spectrum, which confirms the presence of carbon and titanium, indicating successful integration of the nanocomposite components.

The morphology and interactions of the nanocomposite were also studied. The morphology of the wax/polyimide electrode surface before and after the incorporation of CNFs and TiO_2_NPs was characterized using SEM. Figure 4 shows (a) wax surface image, (b) TiO_2_NPs/CNFs/wax at 400×, (c) TiO_2_NPs/CNFs/wax at 800×, and (d) the composition of the nanocomposite TiO_2_/CNFs/wax/polyimide in the EDS spectrum exhibits distinct peaks corresponding to carbon C (Kα, ~0.28 keV), N (Kα, ~0.39 keV), O (Kα, ~0.53 keV), and the main Ti lines (Kα, ~4.51 keV and Kβ, ~4.93 keV), demonstrating the presence of the nanoparticles on the electrode surface.

**Figure 5:**
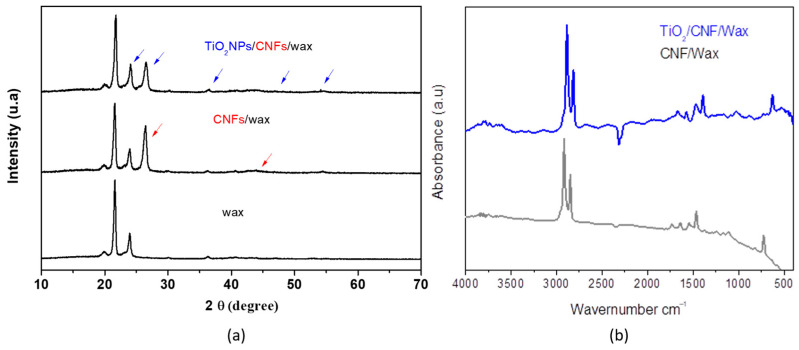
(**a**) shows XRD patterns of wax (black line) and TiO_2_NPs/CNFs/wax (blue line); and (**b**) shows the FTIR spectrum of wax, CNFs/wax and TiO_2_NPs/CNFs/wax.

The crystalline structures of the wax, CNFs/wax, and TiO_2_NPs/CNFs/wax were characterized using an X-ray diffractometer in a range from 10° to 70°. Figure 5a shows that TiO_2_NPs/CNFs/wax presents peaks at 2θ (24.7°, 26.8°, 36°, 48°, and 53.8°), which are attributed to the anatase and rutile TiO_2_ structure [22], and a peak at 2θ (26.8° and 44°) resulting from the (002) crystal plane of graphite crystallites from the CNFs [23]. Meanwhile, wax showed only the peak at 21° and 24°, corresponding to its crystallite structure. In addition, the lower diffraction intensity of TiO_2_NPs/CNFs/wax was observed, suggesting that the TiO_2_NPs/CNFs mixture hinders the formation of the crystal lattice and decreases nanocomposite crystallinity. This decrease in crystallinity may be attributed to the interactions between TiO_2_NPs, CNFs, and the amorphous wax matrix, which can introduce steric hindrance and restrict the regular arrangement of the crystalline components, thereby reducing the long-range order of the nanocomposite structure. Also, TiO_2_NPs/CNFs/wax and CNFs/wax interactions were investigated using FTIR spectroscopy (Figure 5b). The CNFs/wax exhibited bands at 2955 and 2844 cm^−1^, corresponding to the bond stretching vibrations of the CH groups, and a peak at 1733 cm^−1^, attributed to the carbonyl (C=O) stretching vibration from free carboxylic acids and esters [24]. There were also two small bands at 3191.72 and 1627.28 cm^−1^, corresponding to surface-adsorbed water and free hydroxyl groups. In contrast, the TiO_2_NPs/CNFs/wax spectrum presented bands at 460 and 716 cm^−1^, which were ascribed to the vibration and stretching of the Ti-O-Ti linkages in TiO_2_NPs [25]. Additionally, a band in the range of 3800–3400 cm^−1^ was observed, corresponding to the amino groups present in the NPs, and a shift in the carbonyl band (C=O) to 1726 cm^−1^, probably due to interactions between the amino and carboxyl groups of the polymeric matrix.

### 3.2. Optimization

#### 3.2.1. Optimization of Variables Involved in the Construction of the Electrode

The viscosity of the solid ink was investigated at 95 °C. It was observed that the viscosity of the composite solid ink increases primarily with the rise in CNF concentration (being the major component), reaching a maximum at 35% by weight of the wax. Beyond this, percentages of fiber generated significant changes in the viscosity and fluidity of the nanocomposite at the working temperature. This effect is attributed to enhanced interactions between the CNF, which saturate the load capacity of the polymeric matrix.

**Figure 6:**
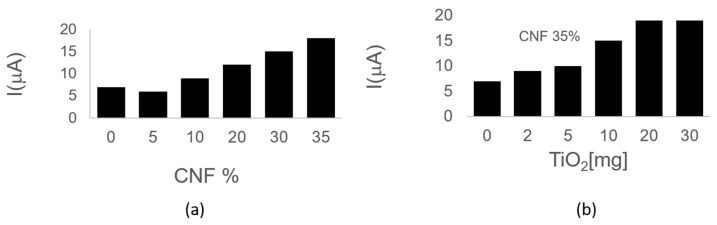
Electrochemical characterization of the nanocomposite inks shows that increasing CNF to a concentration of 35% enhances conductivity (**a**). Similarly, TiO_2_NPs reached maximum electrochemical response at 20 mg (**b**).

The fabrication process comprises stages that can impact the electrochemical performance of the device, including the percentage of the CNF and TiO_2_NPs mixture, the potential, and time applied during the activation process. These factors were electrochemically characterized by cyclic voltammetry (CV) in a 1 mmol [Fe(CN)6]^4−/3−^ redox couple solution in PBS 0.1 mol L^−1^ pH 6.50. Figure 6a illustrates that increasing the CNF concentration enhances the electrochemical signal. As anticipated, the addition of CNF improves the conduction of the wax. However, physical parameters restrict the CNF/wax ratio, limiting the maximum CNFs concentration to 35%. Therefore, a concentration of 35% CNF was selected as the optimal. TiO_2_NPs exhibited their peak electrochemical signal at a concentration of 20 mg (Figure 6b).

The optimization of the activation potential was examined within a range of −1 to −12 V, where an electrochemical signal improvement could be observed at potentials near −9 V (Figure S1a). To optimize the activation time, the electrochemical signal was monitored over a range of 5−100 s, with the current response peaking at 15 s (Figure S1b). Therefore, an activation potential of −9 V and a duration of 15 s were selected for the activation process.

The TiO_2_NPs/CNFs/wax nanocomposite was analyzed under optimal conditions using FTIR, both before and after the activation process. These evaluations revealed no significant changes in the material, suggesting the absence of new covalent bond formation. Thus, it can be concluded that the activation process does not alter the structure of the nanocomposite.

#### 3.2.2. Optimization of Variables Related to the Immunoassay

The TGA enzyme concentration to be immobilized on the surface of the reaction microzone was one of the optimized parameters. In our case, this concentration was evaluated by the HRP saturation method. For this purpose, increasing concentrations of enzyme TGA (1−20 μg mL^−1^) were added to the microzone in different electrodes. Then, a constant and saturating amount of HRP (5 mg in 0.1 mL of PBS 0.01 mol L^−1^ pH 7.2) was added to each microzone electrode. The HRP enzyme adsorbs to the sites not previously occupied by the TGA. The substrate solution containing H_2_O_2_ 1 mmol L^−1^ and Q 1 mmol L^−1^ in 1 mmol L^−1^ citrate-phosphate buffer at pH 5 was added. The HRP in the presence of H_2_O_2_ catalyzes the oxidation of Q to BQ. The electrochemistry reduction in BQ was detected in the surface of TiO_2_NPs/CNFs/wax/polyimide electrode at −0.15 V. Figure S2 shows that the generated current decreases when the concentration of enzyme TGA increases due to less availability of sites for HRP adsorption. Hence, the generated current was inversely proportional to the concentration of immobilized enzyme TGA. The optimal concentration of TGA to immobilize was 5 µg mL^−1^.

**Figure 7:**
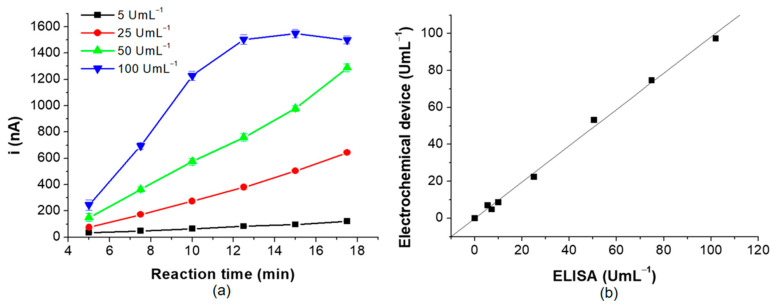
(**a**) shows the optimization of reaction time and (**b**) exposes the correlation between the proposed method and the spectrophotometric ELISA reference method.

Ultimately, the incubation time was evaluated since this represents an essential factor when a reduction in analysis time is required. This parameter was evaluated using four standard solutions containing anti-TGA (5, 25, 50 and 100 U mL^−1^). For concentrations of 5, 25, and 50 U mL^−1^, the signal increased with the rise in the concentration of these antibodies, while for the higher concentration standard (100 U mL^−1^), the current intensity increased until 12.5 min of incubation time. This effect is due to the saturation of the binding sites of the specific antibodies. Therefore, the optimum reaction time was 12.5 min (Figure 7a).

### 3.3. Quantitative Determination of Anti-TGA and Their Application in the Serological Diagnosis of CD

The quantitative determination of anti-TGA IgA type as a part of the serological diagnosis of CD was performed using the designed electrochemical immunosensor in patients’ serum samples. A linear relation was observed, i (nA) = 0.00834 + 0.01502 × Concentration (anti-TGA) (Figure S3), between the current signal and the antibodies concentration in the range of 0 to 100 U mL^−1^. The corresponding correlation coefficient was 0.998. The variation coefficient (CV %) for determining 10 U mL^−1^ anti-TGA antibodies was less than 3.6% (n = 5). The limit of detection (LOD) of the electrochemical device for the analyzed samples was 0.59 U mL^−1^, considering LOD as the concentration that generates a signal 3.29 times the standard deviation of the target on its signal.

The concentration of the anti-TGA standards was also determined through the traditional method of spectrophotometric Enzyme-Linked Immunosorbent Assay (ELISA), obtaining the corresponding calibration curve. The linear regression equation was A = 0.165 +0.026 x Concentration (anti-TGA) with a linear relation coefficient r = 0.991 and a CV% for the determination of 10 U mL^−1^ anti-TGA of 7.8% (n = 5). For the traditional method ELISA, the LD was 1.7 U mL^−1^.

It also evaluated the correlation between the electrochemistry immunosensor and the standard spectrophotometric ELISA method for quantifying IgA type anti-TGA. The slope obtained was close to 1, indicating a good correlation between both methods (Figure 7b).

A relevant analytical factor in the anti-TGA determination is precision. It was evaluated with intra- and inter-assay approaches, performed in quintuplicate using five independent electrodes for each standard concentration of anti-TGA. The intra- and inter-assay CV % obtained for the five replicas using 10, 25, and 100 U mL^−1^ were less than 3.6% and 6.3%, respectively (Table S1). These results expose the satisfactory repeatability and reproducibility of the sensor. 

**Figure 8:**
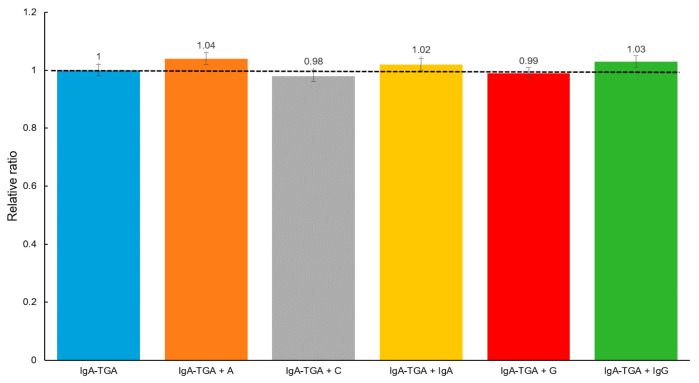
Cross reactivity evaluation: IgA anti-TGA (50 U mL^−1^) detection in presence of albumin (110,000 mg dL^−1^) (A), creatinine (3 mg dL^−1^) (C), immunoglobulin A (700 mg dL^−1^) (IgA), glucose (200 mg dL^−1^) (G), and immunoglobulin G (4000 mg dL^−1^) (IgG).

To evaluate the selectivity of the designed device, a serum sample from the celiac patient, previously tested with a concentration of IgA anti-TGA of 50 U mL^−1^, was analyzed by adding agents present in serum samples, but at concentrations higher than the physiological level (34,000–54,000 mg dL^−1^ for albumin (A), 0.7–1.3 mg dL^−1^ for creatinine (C), 50–400 mg dL^−1^ for immunoglobulin A (IgA), 70–110 mg dL^−1^ for glucose (G), and 700–1600 mg dL^−1^ for immunoglobulin G (IgG), according to the reference values of our country): 110,000 mg dL^−1^ (A), 3 mg dL^−1^ (C), 700 mg dL^−1^ (IgA), 200 mg dL^−1^ (G), and 4000 mg dL^−1^ (IgG). These studies were carried out in quintuplicate using five independent electrodes for each agent evaluated. As shown in Figure 8, in the previously described conditions, the albumin, IgA, and IgG globulins exhibited a 4%, 2%, and 3% of increase in the analytical signal, respectively, while creatinine and glucose displayed an insignificant decrease. At the physiological levels, these agents do not expose interference. These results demonstrate the device’s ability to minimize interferences.

Furthermore, the device’s stability was studied. For this purpose, TiO_2_NPs/CNFs/wax/polyimide electrodes incorporating the recognition agent were assessed. Initially, recently fabricated electrodes (n = 5) were tested and considered as time zero, representing 100% activity. The remaining electrodes were subjected to a lyophilization procedure and stored in a humidity-free container at 4 °C. Each week, for one month, five electrodes (n = 5) were tested and then discarded. The devices showed no significative changes in obtained currents compared to those tested immediately after their fabrication (Figure S4).

### 3.4. Sample Analysis

To show the applicability of the designed electrochemistry device, the concentration of anti-TGA was measured in two control samples provided by the kit, six positive serum samples, and two negatives. In all samples, the presence of normal concentrations of total IgA was verified. Then, they were analyzed using the spectrophotometric ELISA test commonly employed in the clinical field, and finally with the proposed method. The positive samples for the spectrophotometric method were also positive when they were analyzed with the proposed method. The samples that were negative for the traditional test were also negative for the proposed method.

To compare our designed device and its performance with other reported methodologies, the authors perform a bibliographic search. According to this, we could find different methods for IgA anti-TGA determination. Among these, it is essential to mention commercial ELISA kits, which often involve multiple steps, long incubation times, and a large amount of reactive and samples. However, the demand for a fast, sensitive, and selective methodology for serological CD diagnosis has increased. In this sense, and taking into account the antibody concentration units and electrochemical detection techniques, we focus on designed systems for IgA anti-TGA detection, utilizing different immunoassay formats combined with various electrochemical methods [26,27,28,29]. Compared to these, our electrochemical device is the only one that uses flexible electrode support and incorporates a new conductive solid ink based on wax, carbon nanofibers CNFs, and TiO_2_NPs as a platform for the immunorecognition process. The electrochemical detection, performed via amperometry, allowed us to achieve the lowest LOD compared to the methods described in Table 1. Furthermore, the fabricated system requires only 27.5 min for analysis, which is shorter than the time needed for the compared methods (Table S2).

## 4. Conclusions

The search and implementation of new support materials and their adaptation for electrochemistry detection to multiple analytes is an area of constant evolution. In this sense, the polyimide sheets showed to be practical and stable material for their utilization as electrode support. In addition, the incorporation of CNFs and TiO_2_NPs in the wax led to obtaining a solid ink, which was deposited following a three-electrode pattern and exhibited a significant increase in electrochemical signals and immobilization capacity, enabling the quantification of low levels of IgA anti-TGA present in patients with CD.

## Figures and Tables

**Table 1:** Comparison of reported methods for the TGA determination.

Biosensors	Methods	LOD (U/mL)	Linear Range (U/mL)	Analysis Time (min)	Ref.
GC/Polypyrrole-QD	Square wave voltammetry	7.56	0–75	60	[26]
SPCE-MWCNT-NPsAu	Cyclic voltammetry	2.45	0–100	90	[27]
NEEs	Anodic detection	0.7	0.12–123.5	90	[28]
SPCE-CdSe/ZnS QD	Anodic stripping voltammetry	2.7	3–40	80	[29]
Polyimide-wax-CNF-TiO_2_	Amperometry	0.59	0–100	27.5	This work

## Data Availability

Data are contained within the article and the Supplementary Material.

## Supplementary Materials

The following supporting information can be downloaded at: https://www.mdpi.com/article/10.3390/bios15070431/s1, Section S1: The diameter of the working electrode was 3 mm. The Randles–Sevcik equation was used to calculate the electroactive surface area; Figure S1: Optimization of activation potential and activation time; Figure S2: Evaluation of optimal TGA enzyme concentration using the peroxidase enzyme saturation method. Current generated as a function of the concentration of TGA; Figure S3: Calibration curve corresponding to the electrochemical immunosensor designed using standard solutions of anti-tTG antibodies (0–100 U mL^−1^); Figure S4: Stability evaluation; Table S1: Intra-assay precision (five measurements in the same run for each standard); Table S2: Sequences required for the IgA anti-TGA determination.

## Abbreviations

The following abbreviations are used in this manuscript:

CD	Celiac disease
TG	Transglutaminase
anti-TGA	Anti transglutaminase antibody
EMA	Endomysium
ELISA	Enzyme-linked immunosorbent assay
CNF	Carbon nanofiber
NP	Nanoparticle
HRP	Horseradish peroxidase
