# Supplementary material for: A New Sensing Platform Based in CNF-TiO2NPs-Wax on Polyimide Substrate for Celiac Disease Diagnostic
Source: Biosensors (Basel). 2025 Jul 4;15(7):431. doi: 10.3390/bios15070431 (PMC12293545; doi:10.3390/bios15070431)
Supplement: Supplementary file 1 [file biosensors-15-00431-s001.zip › biosensors-3694232-supplementary.pdf]

Supplementary Material

Section S1. Electroactive surface area for the three electrode system designed.

The electroactive surface area of the working electrode for the designed system TiO_2NPs/CNFs/wax/polyimide electrode was calculated by the Randles-Sevcik equation [38]. The diameter of the working electrode was 3 mm. Thus, the surface area was 0.879 cm^2.

Ip = 2.69 × 10 AD n v C

where Ip is the maximum current in amps, A is the electrode area in cm^2, D is the diffusion coefficient in $cm^2\ s^{-1}$, n is the number of electrons transferred in the redox event, ν is the scan rate in V s^{-1}, and C is the concentration in mol L^{-1}.

Figure S1. Optimization of activation potential and activation time.

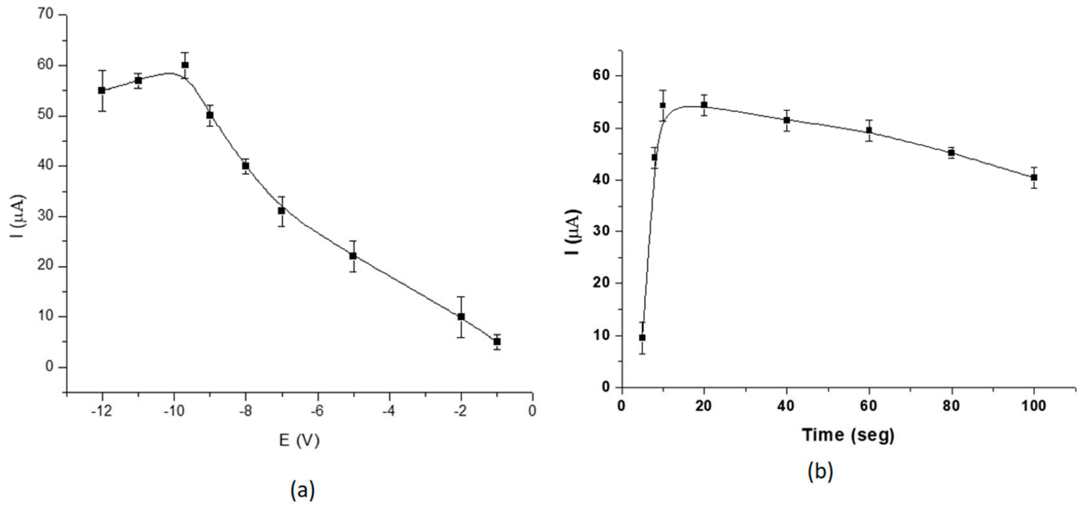

Figure S2. Evaluation of optimal TGA enzyme concentration using the peroxidase enzyme saturation method. Current generated as a function of the concentration of TGA.

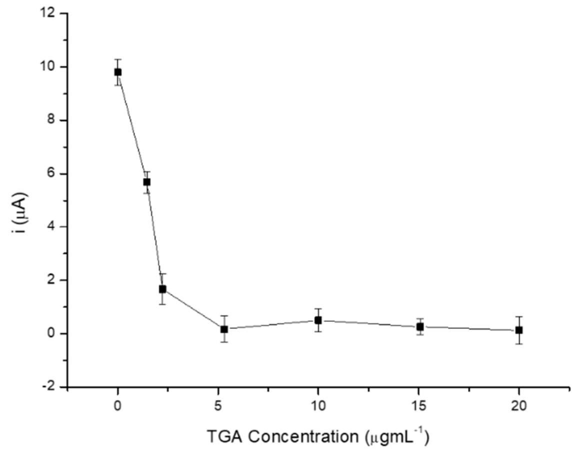

Figure S3. Calibration curve corresponding to the electrochemical immunosensor designed using standard solutions of IgA anti-TGA antibodies (0-100 U mL $^{-1}$).

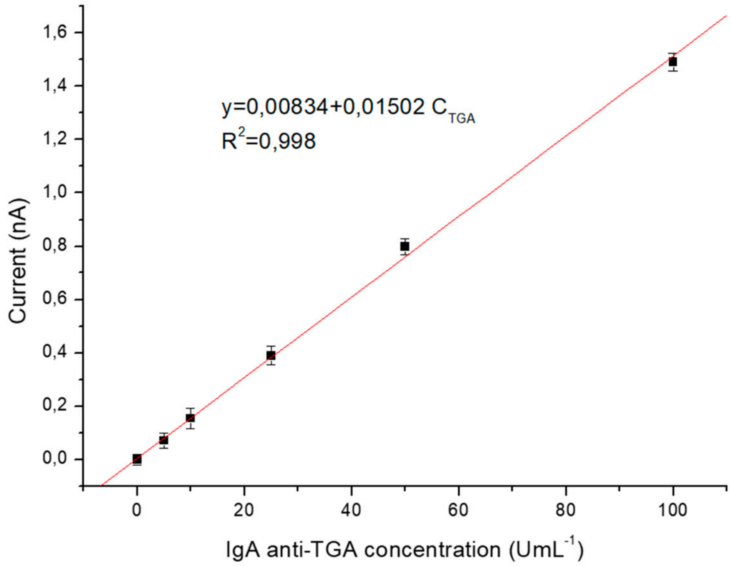

Figure S4. Stability evaluation. TiO_2NPs/CNFs/wax/polyimide electrodes incorporating the recognition agent were assessed. Initially, recently fabricated electrodes (n = 5) were tested and considered as time zero, representing 100% activity. The remaining electrodes were subjected to a lyophilization procedure and stored in a humidity-free container at 4 °C. Each week, for one month, five electrodes (n = 5) were tested and then discarded. The devices showed no significant changes in obtained currents compared to those tested immediately after their fabrication.

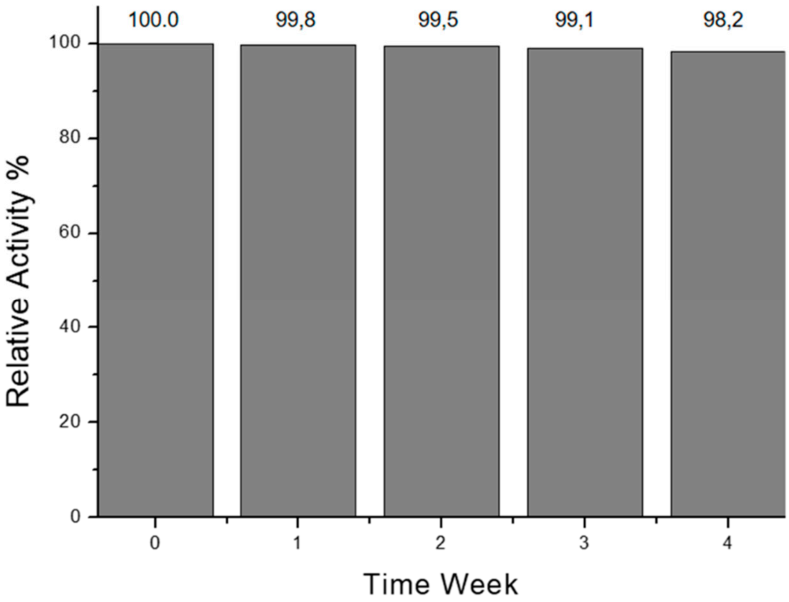

Table S1. Intra-assay precision (five measurements in the same run for each anti-TGA antibody standard solution) and inter-assay precision (five measurements for each anti-TGA antibody standard, repeated for three consecutive days).

Standards	Intra-assay		Inter-assay	
**Anti-TGA**	**Mean**	**CV%**	**Mean**	**CV%**
**5 UmL^{-1}**	4.89	3.31	5.69	5.72
**10 UmL^{-1}**	10.21	3.52	9.75	5.31
**25 UmL^{-1}**	24.81	3.28	25.31	6.13

Table S2. Sequences required for the IgA anti-TGA determination.

Stages	Conditions	Time
**Blocking solution**	BSA al 1% en PBS 0.01 mol L^{-1} pH 7.2	5 min
**Washing buffer**	PBS 0.01 mol L^{-1} pH 7.2	1 min
**Serum samples**	Diluted 100-fold	12.5 min
**Washing buffer**	PBS 0.01 mol L^{-1} pH 7.2	1 min
**Enzymatic conjugated**	HRP-conjugated (dilution of 1/1000)	5 min
**Washing buffer**	PBS 0.01 mol L^{-1} pH 7.2	1 min
**Substrate**	5 μL 1 × 10^{-3} mol L^{-1} H_2O_2 and 1 × 10^{-3} M Q in phosphate citrate buffer, pH 5.0	1 min
**Signal obtention**	LC-4C amperometric detector, 100 mV	1 min
**Total assay time**		27.5 min
